# Translation, Cultural Adaptation, and Validation of the Hiligaynon Montreal Cognitive Assessment Tool (MoCA-Hil) Among Patients With X-Linked Dystonia Parkinsonism (XDP)

**DOI:** 10.3389/fneur.2019.01249

**Published:** 2019-11-28

**Authors:** Nicole B. Aliling, Adovich S. Rivera, Roland Dominic G. Jamora

**Affiliations:** ^1^Department of Neurosciences, Philippine General Hospital, University of the Philippines Manila, Manila, Philippines; ^2^Feinberg School of Medicine, Northwestern University, Evanston, IL, United States; ^3^Department of Neurosciences, College of Medicine—Philippine General Hospital, University of the Philippines Manila, Manila, Philippines; ^4^Movement Disorder Service, St. Luke's Medical Center, Institute for Neurosciences, Quezon City, Philippines

**Keywords:** XDP, X-linked dystonia parkinsonism, cognitive impairment, montreal cognitive assessment (MoCA), hiligaynon, MoCA-Hil, validation

## Abstract

**Background:** X-linked dystonia parkinsonism (XDP) is a neurodegenerative disease endemic to Filipinos with maternal lineage from Panay Island, Philippines. Patients present with dystonia concurrent with or followed by parkinsonism. Non-motor symptoms also predominate, affecting behavior and cognition. We aimed to translate and do cross-cultural adaptation and validation of the Montreal Cognitive Assessment Tool (MoCA) into Hiligaynon (MoCA-Hil), the language spoken in Panay Island, to perform baseline cognitive screening of XDP patients.

**Methods:** Forward translation to Hiligaynon was done by two translators, then back translation of a single version was adapted and approved by a committee. A pilot testing was done yielding the final translated version, which was then tested on 46 XDP patients. The test-retest reliability was measured for 11 patients. The XDP-MDSP (Movement Disorder Society of the Philippines) rating scale was used to assess disease severity.

**Results:** The MoCA-Hil showed an acceptable test-retest reliability [intraclass correlation (ICC) 0.74] and internal consistency (Cronbach's alpha 0.86 at baseline, 0.81 at 12 weeks). The two subscales with low ICC at 0.09 and 0.21 were delayed recall and orientation, respectively.

**Conclusion:** Translation, cultural adaptation and validation of the MoCA to Hiligaynon was successfully done. This tool may now be used in clinical practice and in research for Hiligaynon-speaking subjects.

## Introduction

X-linked dystonia parkinsonism (XDP) is a degenerative neurologic disease endemic to Filipinos whose maternal lineage is from the provinces of Capiz, Iloilo, Aklan, and Antique in Panay Island, Philippines. It is a rare condition—as of 2010, XDP was detected in 0.31 per 100,000 of the Philippine population and 5.74 per 100,000 in Panay Island (1). The motor features usually begin as a focal dystonia that generalizes in 2–5 years, then coincides with or is replaced by parkinsonism after 10 years (1).

The pathology in patients with XDP includes atrophy and astrogliosis of the caudate head and putamen and reduction of striatopallidal and striatonigral projections. Neuronal loss in the corpus striatum striosome compartment responsible for modulating nigral dopaminergic functions was theorized to cause the initial symptoms of dystonia, while the later involvement of the striatal matrix may explain the development of parkinsonism (2, 3).

Apart from its manifestations as a movement disorder, XDP also presents with non-motor symptoms. A review of limited available data showed that 76% of patients had impairments in the frontal lobe functions of abstract thinking and motor planning/executive control (4). Mood disorders, such as depression and anxiety, were also observed among XDP patients. These may occur concurrently with cognitive impairment or may mislead to a diagnosis of dementia in cognitively intact individuals (4).

The Montreal Cognitive Assessment Tool (MoCA) is known as an effective screening questionnaire to assess mild cognitive impairment (5). Its validity as a screening tool for cognitive dysfunction and its superiority over the mini-mental state exam (MMSE) have been demonstrated, not only in mild cognitive impairment but also in the setting of Alzheimer disease, stroke, vascular dementia, traumatic brain injury and frontotemporal dementia. The usefulness of the MoCA as a suitable, accurate and brief test for screening all levels of cognition in Parkinson's disease (PD) has also been established (6).

This tool has been validated in the national language of the Philippines, Filipino (MoCA-P) (7, 8). Thus, in order to account for the patient's linguistic and ethnic background and to properly conduct a baseline assessment of cognitive function among XDP patients in Panay Island, we aim to translate and validate the MoCA to Hiligaynon (MoCA-Hil), while maintaining the psychometric properties of the original English version and adapting the applicable cultural considerations of the MoCA-P.

## Methods

### Study Design

A cross sectional analytic study design was employed. Permission to adapt the MoCA was obtained from Prof. Ziad Nasreddine, the primary author of the MoCA.

### Target Population, Subject Sampling, Sample Size Calculation

The study participants for the pilot test were 40 healthy adult males whose native language was Hiligaynon, but were also fluent in Filipino and English. For the pre-test and validation, a consecutive sample of 46 genetically confirmed XDP patients hailing from or with roots traced to Panay Island were recruited. Likewise, these XDP patients use Hiligaynon as their primary language but were also articulate in Filipino and English. We excluded clinically diagnosed XDP patients without genetic test results.

### Procedure

The translation and cross-cultural adaptation process of the MoCA-Hil was patterned after published guidelines and good practice principles (9–11).

#### Forward Translation

The forward translation of the MoCA to Hiligaynon was done by two independent translators [Translations 1 and 2 (T1 and T2)] who are native speakers of Hiligaynon, English and Filipino. One translator was commissioned from the *Sentro ng Wikang Filipino* (Center for the Filipino Language, Nora A. Garde) of the University of the Philippines Diliman, who had no prior knowledge of the cognitive assessment tool or medical background. Another translator is a clinician currently practicing in Roxas City, Capiz (Glenn L. Grecia, Jr.), who is familiar with the concepts tested by the MoCA.

The English version was used as the basis for the translation in order to avoid compounding errors or biases if the Filipino version (7) was used as the source text. Culturally relevant adaptations to Hiligaynon include using words for the memory subscale that stood for face, blue, church, rose, and silk (instead of face, velvet, church, daisy, and red in the English version of MoCA) and replacing the rhinoceros placed under the naming test was with an owl. The word fluency test in the language subscale was also changed from the letter F to letter B. These cultural adaptations were deemed acceptable by the Hiligaynon linguists.

#### Synthesis of Translation 1 and Translation 2 to Translation 3

A committee comprised of the authors and one of the forward translators then reviewed the forward translations (T1 and T2) and came up with a consensus version, translation 3 (T3).

#### Back Translation

A third independent translator, a clinician who is a native speaker of Hiligaynon, English and Filipino (Carmelo Adrian B. Almeida), who was blinded to the original version of the MoCA, rendered the back translation from Hiligaynon to English.

#### Committee Review

The committee reviewed all translated versions (T1, T2, T3, and back translation) and the reports regarding reaching a consensus for T3 and about comparing the original questionnaire and back translated tool. The Hiligaynon expert from the *Sentro ng Wikang Filipino* (Center for the Filipino Language) of the University of the Philippines Diliman also prepared the instructions for conduct of the interview apart from the translated MoCA-Hil tool. This resulted in the pre-final version of the MoCA-Hil.

#### Test of the Pre-final Version

The pre-final version of the MoCA-Hil was pilot tested among 40 healthy male subjects who were fluent in English, Hiligaynon, and Filipino. This sample size was determined by rule of thumb, which suggests 30–40 persons should be tested to evaluate the psychometric properties of a measurement instrument (11). All study subjects completed a demographic questionnaire. Afterwards, the subjects underwent an interview using the pre-final version of the MoCA-Hil, which took an average of 15 min to complete. The interview was performed by a clinician based in Roxas City, Capiz, who was trained to use the tool by the primary author (NBA). Upon completion, the subjects were asked whether there were difficulties encountered in the conduct of the interview or if further clarifications were needed. These were included in the report submitted to the committee.

#### Committee Appraisal

The final version of MoCA-Hil (Supplementary Figure 1) was developed based on the result of the pilot testing and the written reports of the interviewers and translators, as agreed upon with the authors. The instructions in Hiligaynon for the conduct of the MoCA-Hil was also prepared by the *Sentro ng Wikang Filipino* (Center for the Filipino Language) of the University of the Philippines Diliman (Supplementary File).

#### Test-Retest Reliability and Validation

The pre-testing of the final iteration of the MoCA-Hil was done on 46 male XDP patients, following the aforementioned rule of thumb applied for the healthy participants. The validation of the tool was done on XDP patients, who comprised the target population (11). Other non-XDP parkinsonian patients were excluded from the study to avoid diluting the homogeneity of the group. The test-retest reliability of the tool was measured, comparing initial scores to repeat interviews done after 12 weeks. Interviews were performed by the initial interviewer who did the pilot testing, as well as a genetic counselor based in Roxas City, Capiz. Both interviewers were trained on using the MoCA-Hil tool by one of the authors (NBA).

Descriptive data, including age in years, duration of illness, their stage of disease and degree of functional impairment was gathered. The severity of symptoms and functional disability was quantified using the XDP-MDSP scale developed by the Movement Disorder Society of the Philippines (MDSP) (12).

The content validity of the MoCA and MoCA-P versions have previously been established and is beyond the scope of this study. Likewise, construct validity could not be measured as there are no other validated neurocognitive scales available in the Hiligaynon language that may be used for comparison.

### Statistical Analysis

Data analysis was performed in Stata SE version 13. The reliability analyses of internal consistency of the MoCA-Hil was computed using Cronbach's alpha, to measure the degree to which items that make up the total score are all measuring the same underlying attribute. A value of ≥ 0.70 is acceptable. The test-retest reliability was assessed using intraclass correlation coefficients (ICC). Values of ≥ 0.5 are considered good (11). The relationships between MoCA-Hil subscales to the XDP-MDSP subscale scores and other variables were analyzed using the Pearson's correlation coefficient.

### Ethical Considerations

This study was carried out in accordance with the recommendations of the University of the Philippines Manila Research Ethics Board (UPMREB). The protocol was approved by the UPMREB (NEU) 2017-328-01. All subjects gave written informed consent in accordance with the Declaration of Helsinki.

## Results

The mean age of the 40 healthy male participants who participated in the pilot testing of the pre-final version of the questionnaire was 36.05 ± 13.39 years. The length of formal education was 11.65 ± 4.05 years.

The mean age of the 46 male XDP patients interviewed was 50.2 ± 9.2 years. The mean length of formal education was 10.4 ± 3.7 years equating to secondary/ high school level as the highest educational attainment. The difference between the mean age of the healthy controls and the XDP patients reached statistical significance (Table 1).

**Table 1:** Demographics of subjects.

	**Healthy controls (*n* = 40)**	**XDP patients (*n* = 46)**	***p*-value**
Age, in years (Mean ± SD)	36.1 ± 13.4	50.2 ± 9.2	<0.001
Level of education (%)			0.597
College/Vocational	19 (47.5%)	22 (47.8%)	
High school	15 (37.5)	11 (23.9%)	
Elementary	6 (15%)	13 (28.3%)	
Years of formal education (Mean ± SD)	11.7 ± 4.1	10.4 ± 3.5	0.145

The majority of XDP patients seen (*n* = 29, 63%) were in stage III of the disease, with a mean duration of illness of 6.8 ± 7.0 years. The mean score on the XDP-MDSP rating scale was 49.3 ± 22.8 out of a possible maximum score of 200. The medications used was also summarized, as well as the number of those who underwent deep brain stimulation. These baseline characteristics (Table 2) were noted in order to assimilate their performance in the MoCA test with possible confounding factors not directly related to their level of cognition.

**Table 2:** Profile of XDP patients.

**Variable**	***N* (%)**
**Stage of disease**	
I	4 (8.7)
II	6 (13.0)
III	29 (63.0)
IV	7 (15.2)
V	0
Duration of disease, in years (mean ± SD)	6.8 ± 7.0
XDP-MDSP total score (mean ± SD)	49.3 ± 22.8
**Drugs used**	
Biperiden	39 (84.8)
Clonazepam	34 (73.9)
Levodopa	17 (37.0)
Botulinum toxin injection	14 (30.4)
Zolpidem	5 (10.9)
Piribedil	1 (2.2)
Ropinirole	1 (2.2)
Deep brain stimulation	5 (10.9)

### MoCA-Hil Scores of XDP Patients

The mean MoCA-Hil total score of the XDP patients was 19.2 ± 4.3, compared to the higher scores of control subjects, 24.5 ± 4.1 (*p* < 0.001). The specific areas of difficulty pertained to tests of language, abstraction, attention and visuospatial skills. Higher scores were garnered for orientation, naming, delayed recall and serial seven subtraction. Using the cut-off score of 21 from the validated Filipino version, 25 (54.3%) of the XDP patients tested may have cognitive impairment, vs. only 7 (17.5%) of the healthy subjects (Table 3).

**Table 3:** MoCA-Hil results of subjects.

	**Healthy controls (*n* = 40)**	**XDP patients (*n* = 46)**	***p*-value**
**MoCA-Hil**	**Mean ± SD**	**Mean ± SD**	
Adjusted score^+^	24.5 ± 4.1	19.2 ± 4.3	<0.001
Raw score	24.1 ± 4.5	18.8 ± 4.6	<0.001
**Subscale scores**			
Visuospatial/Executive	4.0 ± 1.2	2.8 ± 1.5	<0.001
Naming	2.7 ± 0.6	2.4 ± 0.8	0.080
Attention	2.4 ± 0.8	1.6 ± 1.0	<0.001
Serial sevens	2.6 ± 0.6	1.8 ± 1.1	<0.001
Language	1.2 ± 0.9	0.5 ± 0.8	0.001
Abstraction	1.2 ± 0.7	1.0 ± 0.6	0.209
Delayed recall	4.3 ± 0.9	3.0 ± 1.4	<0.001
Orientation	5.8 ± 0.6	5.6 ± 0.7	0.236
Cognitive impairment (≤20) [*n* (%)]	7 (17.5)	25 (54.3)	<0.001

+ *Additional two points are added to the raw score for those with ≤7 years of formal education, while one point is added for those with 8–12 years of formal education. For those with more than 12 years of formal education, no adjustment was done*.

### Internal Consistency

The instrument had good internal consistency with a Chronbach's alpha of 0.86 during the initial test. The Cronbach's alpha of the XDP patients' results was 0.79. Similar results were seen during the re-test of the subjects, with a Chronbach's alpha at 0.81, 0.79, and 0.85 for all subjects, XDP patients and healthy controls, respectively (Table 4).

**Table 4:** Cronbach's alpha coefficient on initial and re-test.

	**Initial*^+^***	**Re-test*^+^***
Combined	0.86	0.81
XDP patients	0.79	0.79
Healthy controls	0.86	0.85

+ *Adjusted scores were used for analysis. Additional two points are added to the raw score for those with ≤ 7 years of formal education, while one point is added for those with 8–12 years of formal education. For those with more than 12 years of formal education, no adjustment was done*.

### Test-Retest Reliability

Retesting was done in 11 patients who were able to follow up. The MoCA-Hil exhibited good test-retest reliability among XDP patients, with comparable initial and retest mean scores and an ICC of 0.74 (95% CI: 0.49–0.88) for the total scores adjusted for the number of years of formal education (Table 5). The mean difference in the adjusted total score was −1.45 ± 2.2. The ICC for each subscale ranged from 0.09 to 0.75. The delayed recall and orientation were the two subscales with low ICC at 0.09 (−0.31 to 0.46) and 0.21 (−0.20 to 0.56).

**Table 5:** Test-retest reliability of the MoCA-Hil among XDP patients.

**Scale**	**ICC**	**95% confidence interval**	***p-*value**
Adjusted score	0.74	0.49 to 0.88	<0.001
Raw score	0.77	0.55 to 0.89	<0.001
**Subscales**			
Visuospatial/ Executive	0.52	0.16 to 0.76	0.003
Naming	0.72	0.47 to 0.87	<0.001
Attention	0.75	0.51 to 0.88	<0.001
Serial sevens	0.70	0.43 to 0.86	<0.001
Language	0.61	0.29 to 0.81	<0.001
Abstraction	0.60	0.27 to 0.8	0.001
Delayed recall	0.09	−0.31 to 0.46	0.337
Orientation	0.21	−0.2 to 0.56	0.153

### Correlation of MoCA-Hil Scores With Clinical Features and the XDP-MDSP Rating Scale Scores

XDP patients were grouped according to stages of disease to compare their performance on the MoCA-Hil using ANOVA. The mean total and subscale scores among the four stages were not significantly different (Table 6).

**Table 6:** Mean and significance level of MoCA-Hil subscales at different stages of disease.

**Scale**	**Stages (Mean ± SD)**				
	**I (*n* = 4)**	**II (*n* = 6)**	**III (*n* = 29)**	**IV (*n* = 7)**	***p-*value**
Adjusted score	22.0 ± 4.3	18.5 ± 5.4	19.7 ± 4.0	16.0 ± 3.3	0.099
Raw score	21.5 ± 5.3	17.8 ± 6.0	19.5 ± 4.2	15.4 ± 3.8	0.110
**Subscales**					
Visuospatial /Executive	3.8 ± 0.5	2.5 ± 1.6	3.1 ± 1.5	1.6 ± 1.3	0.056
Naming	2.8 ± 0.5	2.8 ± 0.4	2.4 ± 0.7	1.9 ± 1.2	0.104
Attention	2.0 ± 1.4	1.5 ± 1.4	1.7 ± 1.0	1.1 ± 0.9	0.512
Serial sevens	2.0 ± 1.2	1.5 ± 1.2	2.0 ± 1.1	1.3 ± 0.8	0.431
Language	0.3 ± 0.5	0.8 ± 1.0	0.6 ± 0.8	0.3 ± 0.5	0.529
Abstraction	1.0	0.8 ± 0.8	1.0 ± 0.6	1.0 ± 0.6	0.911
Delayed recall	4.0 ± 1.4	2.5 ± 1.5	3.1 ± 1.4	2.4 ± 1.4	0.277
Orientation	5.8 ± 0.5	5.3 ± 1.2	5.6 ± 0.7	5.9 ± 0.4	0.603

The factors contributing to the performance of the XDP patients on the MoCA-Hil are shown in Table 7. A higher MoCA-Hil scores was correlated with more years of formal education, while a lower MoCA-Hil score was seen with increasing age. The duration of XDP disease duration did not have a significant linear relationship with the MoCA-Hil scores.

**Table 7:** Correlation of the MoCA-Hil subscales and clinical features and the XDP-MDSP scale using Pearson's r.

**Scale**	**Age**	**XDP duration**	**Formal education duration**	**XDP-MDSP Scale**					
				**I**	**II**	**III**	**IV**	**V1**	**Total**
Adjusted score*^+^*	−0.53^**^	0.07	0.45^*^	−0.36^*^	−0.27	−0.32^*^	−0.12	−0.27	−0.34^*^
Raw score	−0.56^**^	0.03	0.55^**^	−0.34^*^	−0.26	−0.34^*^	−0.13	−0.27	−0.34^*^
**Subscales**									
Visuospatial/Executive	−0.55^**^	−0.17	0.55^**^	−0.30^*^	−0.34^*^	−0.44^*^	−0.25	−0.40^*^	−0.42^*^
Naming	−0.20	0.08	0.24	−0.16	−0.18	−0.17	0.01	−0.01	−0.16
Attention	−0.50^**^	−0.12	0.74^**^	−0.21	−0.15	−0.26	−0.01	−0.12	−0.19
Serial sevens	−0.18	0.36^*^	0.14	−0.16	0.20	−0.11	0.02	0.02	0.01
Language	−0.33^*^	−0.09	0.32^*^	−0.10	−0.11	−0.01	0.08	0.04	−0.06
Abstraction	−0.11	−0.13	0.15	−0.09	−0.10	−0.20	−0.16	−0.32	−0.17
Delayed recall	−0.37^*^	0.03	0.15	−0.31^*^	−0.31^*^	−0.18	−0.10	−0.21	−0.29^*^
Orientation	−0.07	0.24	0.10	−0.05	−0.01	−0.03	−0.10	−0.17	−0.06

* *p < 0.05*.

** *p < 0.001*.

+ *Additional two points are added to the raw score for those with ≤ 7 years of formal education, while one point is added for those with 8–12 years of formal education. For those with more than 12 years of formal education, no adjustment was done*.

There was a weak negative correlation between XDP-MDSP rating scale total score and the MoCA-Hil scores. The MoCA-Hil scores were also weakly correlated with the XDP-MDSP parts I and III. No linear relationship was observed between MoCA-Hil score and XDP-MDSP parts II, IV and VI. The relationship between MoCA-Hil subscales to the XDP-MDSP rating scale scores were also examined. There was a negative correlation between the scores on the visuospatial/executive part of the MoCA-Hil to the XDP-MDSP rating scale scores.

## Discussion

The translation, cross-cultural adaptation and validation of the MoCA-Hil tool was successfully done by following published guidelines (9–11). The MoCA-Hil was shown to have good internal consistency, with a Cronbach's alpha coefficient of 0.86. This was lower compared to the MoCA-P (Cronbach's alpha coefficient of 0.938) (7), but similar to the alpha coefficient of the original MoCA instrument in English, which was 0.83 (5). The test-retest reliability was acceptable, with an ICC of 0.74 (95% CI: 0.49–0.88). This was however lower than the English version (correlation coefficient 0.92, *p* < 0.001) (6). The mean difference of the adjusted total score was −1.45 ± 2.2, almost twice that of the mean change seen among the elderly patients tested for MoCA-P (−0.76 ± 2.26) (7).

On subscale analysis, delayed recall and orientation were the two subscales with low ICC at 0.09 (−0.31 to 0.46) and 0.21 (−0.20 to 0.56), respectively. This reflects high variability of the scores in these areas between tests. Orientation was again a weakly reliable measure in terms of item-rest correlation measurement during the retest, making it the least reflective subscale of a patient's performance in the MoCA-Hil. A high drop-out rate (*n* = 35, 76%) among the subjects who participated in the retest, may explain this discrepancy.

Further investigation of the content validity of each of the items in the MoCA-Hil was no longer done, as the original English and the translated Filipino versions where this iteration was derived from were already validated. We acknowledge that this step is suggested in some studies (11). The cultural validity of certain items when translated to Hiligaynon, such as using the letter “B” for the language subscale, was verified with the translators.

The level of education is an important determinant of the degree of cognitive impairment. The average educational attainment of the XDP patients was until secondary schooling, equivalent to 10 years of education. This factor was addressed in the validation of the MoCA-P, wherein a lower cut-off of 20 was set to discriminate between early stage probable dementia and control subjects (8). This will yield a sensitivity of 83.5% and specificity of 72.3%. The researchers also accounted for low education, defined as seven or less years of formal schooling. As such, we adapted the recommended practice of adding two points to the MoCA score for those with seven or lower years of education, and adding one point for those with 8–11 years of schooling. With this adjustment, the MoCA-P authors recommended using a single cut-off score at 22 (sensitivity 89.7%, specificity 54.2%), as was done in this research (8).

Preliminary data among XDP patients included in this research shows that as high as 54.3% (25 of 46 patients) have scores that are suspect for probable dementia in a relatively younger age group (mean age of 50.2 ± 9.2 years). In a previous review, 76% (16 of 21) patients aged 47 ± 9.03 years had documented cognitive impairment in at least one of the three administered screening tests (4). These tests, the MMSE, clock-drawing test (CDT) and frontal assessment battery (FAB), all have components that are similarly represented by the MoCA. Our results showed that the areas of greatest deficit were language, abstraction, attention and visuospatial skills. In the same review mentioned, attention and calculation, orientation, language were the subscales that garnered the most errors (4). In the CDT of this review and in the visuospatial skills part of the MoCA-Hil, most patients who failed the test had difficulty placing the correct time in the clock. Test performance in abstraction or similarities, as well as lexical fluency and word retrieval, were problem areas in this review (4).

Neurodegenerative pathologic and neuroimaging correlates posed theories on the development of these cognitive symptoms in XDP, exemplified by related disorders. Reports in DYT1 patients point to a possible selective dysfunction in the cortical-frontal-basal ganglia-thalamus-cortical network, explaining these deficits (13). In PD dementia, impaired problem solving and disorganized planning comprise a frontal dysexecutive syndrome (14). Huntington's disease, which shows similar findings in XDP such as caudate atrophy, also presents with dysfunction of attention and executive function (15). In addition, positron emission tomography results in XDP showed selective reduction in striatal glucose metabolism and loss of striosomal and matrix-based inhibitory projections from the neostriatum (2, 3, 16).

These results were examined on the background of the motor and non-motor features of XDP, that directly affect patients' ability to perform certain tasks required in screening tests. The disease severity was measured with the use of the novel scale developed specifically for XDP, the XDP-MDSP Rating Scale. It is a composite of different tools encompassing the varied manifestations of XDP: the Burke-Fahn-Marsden dystonia rating scale for dystonia, the Unified PD Rating Scale motor subscale for parkinsonism, the Non-Motor Symptoms Questionnaire for non-motor symptoms, and the Short Parkinson's Evaluation Scale/ Scales for Outcomes in PD for motor impairments, activities of daily living, and motor complications (12).

A regression analysis of our data revealed a weak correlation between the total scores of the MoCA-Hil and the XDP-MDSP rating scale. An inverse linear relationship was established, such that better performance in the MoCA-Hil was associated with a milder disease presentation. This linear relationship was demonstrated in XDP-MDSP rating scale parts I and III, corresponding to the dystonia and non-motor features subscales, respectively. One of the items asked in the non-motor subscale pertains to cognition, explaining the correlation. Of the 25 patients with probable dementia (MoCA-Hil score of <20), 10 patients reported having symptoms related to memory or attention, denoting the need to examine these scoring systems concurrently while also delineating whom among the patients will benefit from more thorough neuropsychological evaluation and possible therapeutic intervention. Meanwhile, the relationship between the dystonia severity and lower MoCA-Hil score may be explained by physical hindrance from speaking or writing due to dystonic symptoms. There was no linear relationship demonstrated between the MoCA-Hil and the XDP-MDSP rating scale sections on parkinsonism, activities of daily living or global severity.

The XDP patients included in this study were on various combinations of medications. These were noted, as treatment-related factors affecting cognition must also be considered. Anticholinergic agents and benzodiazepines were alluded to possibly affect cognition, but direct causation was not established (13). Indeed, in a case report of a 67 year-old man with a 10-years history of XDP, stopping the intake of trihexyphenidyl and lorazepam did not significantly alter the result of his neuropsychiatric evaluation (17). Alternatively, botulinum toxin injections for the improvement of dystonic activity were seen to positively affect attentional processing, perhaps by decreasing disabling symptoms such as pain (13).

A limitation in this study include the failure to recruit age-matched controls, precluding a fair comparison of their performance to the XDP patients, whose mean age was significantly greater. The lower scores in the MoCA-Hil of XDP patients were likely attributable to this age difference and the role of their disease as an independent factor cannot be ascertained without bias. Retest of all healthy controls and XDP patients was also not completed due to logistical challenges (distance, infrequent, and expensive transportation) making it difficult for the patients to return to the testing sites. The other issues encountered were the inherent disadvantage to timed testing of patients who have speech difficulties or even rendered anarthric due to their jaw opening dystonia—which was seen in four of the subjects. In addition, as the validation process was geared toward developing a tool for use among XDP patients, and as female XDP patients are rare, the MoCA-Hil was not validated among women and is biased toward Hiligaynon-speaking males.

## Conclusions

The MoCA-Hil version has good internal consistency and an acceptable test-retest reliability. It followed a rigorous and stepwise translation of psychometric tools which included safeguards to ensure cultural adaptability. This tool may now be used in routine clinical practice to monitor patient outcomes or for research purposes in patients who speak Hiligaynon.

We also reported preliminary results that are in concordance with previous findings of cognitive impairment in XDP patients. Despite having a relatively young group (mean age 50.2 ± 9.2), 54.3% (*n* = 25/46) patients yielded scores below the cut-off for probable dementia. This study, albeit consisting of a small number of patients and lacking an age-matched control group, has laid the groundwork for a more comprehensive screening for cognitive impairment among XDP patients. Future research should include a more thorough report of their cognitive profiles and an in depth analysis of the factors contributing to their early cognitive decline.

## Data Availability Statement

The raw data supporting the conclusions of this manuscript will be made available by the authors, without undue reservations, to any qualified researcher.

## Ethics Statement

This study was carried out in accordance with the recommendations of the University of the Philippines Manila Research Ethics Board (UPMREB). The protocol was approved by the UPMREB (NEU) 2017-328-01. All subjects gave written informed consent in accordance with the Declaration of Helsinki.

## Author Contributions

NA: study concept and design, acquisition of data, analysis and interpretation, writing of the initial draft, and critical revision of the manuscript. AR: study concept and design, analysis and interpretation, and critical revision of the manuscript. RJ: study concept and design, acquisition of data, analysis and interpretation, critical revision of the manuscript for intellectual content, and study supervision.

### Conflict of Interest

NA and AR declare that they do not have any disclosures. RJ serves on the advisory boards of Lundbeck Phils. and Torrent Phils. He has received honoraria from the Philippine offices of Allergan, Innogen, Lundbeck, Medichem, Natrapharm, Sandoz, Sun, Torrent, and Zydus. He has ongoing researches from the Collaborative Center for X-linked Dystonia-Parkinsonism and St. Luke's Medical Center – Quezon City.

## Supplementary Material

Supplementary Figure 1 Montreal Cognitive Assessment—Hiligaynon (MoCA-Hil).

Supplementary File MoCA-Hil Instructions in Hiligaynon.
