# Corrigendum: Translation, Cultural Adaptation, and Validation of the Hiligaynon Montreal Cognitive Assessment Tool (MoCA-Hil) Among Patients With X-Linked Dystonia Parkinsonism (XDP)

**DOI:** 10.3389/fneur.2020.00947

**Published:** 2020-09-02

**Authors:** Nicole B. Aliling, Adovich S. Rivera, Roland Dominic G. Jamora

**Affiliations:** ^1^Department of Neurosciences, Philippine General Hospital, University of the Philippines Manila, Manila, Philippines; ^2^Feinberg School of Medicine, Northwestern University, Evanston, IL, United States; ^3^Department of Neurosciences, College of Medicine—Philippine General Hospital, University of the Philippines Manila, Manila, Philippines; ^4^Movement Disorder Service, St. Luke's Medical Center, Institute for Neurosciences, Quezon City, Philippines

**Keywords:** XDP, X-linked dystonia parkinsonism, cognitive impairment, montreal cognitive assessment (MoCA), hiligaynon, MoCA-Hil, validation

In the original article, there was a mistake in ***Supplementary Figure 1 and Supplementary File*** as published. **The MoCA-Hil instructions were erroneously published as Supplementary Figure 1. This is supposed to be the MoCA-Hil tool. MoCA-Hil is under copyright of Z. Nasreddine MD and is reproduced with permission. Copies are available at** **www.mocatest.org**. **In addition, the published MoCA-Hil instructions which appear under *Supplementary File* did not follow the prescribed format for reproduction**. The corrected ***Supplementary Figure 1 and Supplementary File*** have been published.

The authors apologize for this error and state that this does not change the scientific conclusions of the article in any way. The original article has been updated.

