# Supplementary material for: Translation, Cultural Adaptation, and Validation of the Hiligaynon Montreal Cognitive Assessment Tool (MoCA-Hil) Among Patients With X-Linked Dystonia Parkinsonism (XDP)
Source: Front Neurol. 2019 Nov 28;10:1249. doi: 10.3389/fneur.2019.01249 (PMC6892978; doi:10.3389/fneur.2019.01249)
Supplement: Supplementary File — MoCA-Hil Instructions in Hiligaynon. [file Data_Sheet_1.pdf]

Montreal Cognitive Assessment Version 7.1 – Hiligaynon (MoCA-Hil)

Instruksiyon Kung Paano Gamiton Kag Paghatag Sang Puntos Ang MoCA-Hil

Ang Montreal Cognitive Assessment – Hiligaynon Version (MoCA-Hil) ay isa ka instrumento nga ginagamit para madasig nga mahibal-an kung ang isa ka sabdyek ay may kalulo o mild cognitive dysfunction. Ang ini nga instrumento ay ginagamit sa pagsukat sang mga cognitive domains sang isa ka sabdyek: atensyon kag konsentrasyon, ehekutibo nga pagpanghikot, memorya, lenguahe, visuoconstructional skills, konseptuwal nga paghunahuna, kalkulasiyon, kag oryentasyon. Na-estimate nga 10 ka minutos ang kalawigon sang pag gamit sang MoCA-Hil. Ang pinakadako nga puntos nga pwede makuha sang isa ka sabdyek sa MoCA-Hil ay 30. Ang normal nga sabdyek ay makakakuha sang puntos nga 22 o mas mataas pa.

1. <u>Pag-ubra Sang Trail [Alternating Trail Making]</u>

<u>Instruksyon sa sabdyek</u>: Tudluan sang manugsayasat ang sabdyek: *"Maghimo sang kurit masugod sa kag pakadto sa numero sa letra nga pasaka nga pagkaareglo. Halin diri* [itudlo ang numero (1)] *kag magkurit sang linya halin sa 1 pakadto sa A pakadto sa 2 kag tubtub sa kaapysan. Diri matapos* [itudlo ang letra (E)].

<u>Instruksyon sa paghatag sang puntos</u>: Hatagan sang isa ka puntos kun madinalag-on nga nahimo sang sabdyek ang mga kurit sa sumusunod nga padron: 1 − A − 2 − B − 3 − C − 4 − D − 5 − E, basta wala sang bisan ano nga linya nga ginkurit ang nakalaktud. Ang bisan ano nga sayop nga indi dayon natadlong sang kaugalingon 0 ang makuha nga puntos.

2. <u>Pag-Kopya sang Drowing (nga Cube) [Visuoconstructional Skills (Cube)]</u>

<u>Instruksyon sa sabdyek</u>: Mahatag ang manugsayasat sang sumusunod nga instruksiyon, nga ginatudlo ang **kubo**: *"Kopyahon mo ang drowing sing tubtub nga imo masarangan sa lang-at nga ara sa idalom."*

<u>Instruksyon sa paghatag sang puntos</u>: Kung tama ang pag-kopya sang sabdyek sang drowing nga cube, hatagan ang sabdyek sang isa ka puntos.

- Importante gid nga ang drowing ay may tatlo ka dimensyon.
- Dapat kumpleto ang mga kurit
- Wala sang gin-dugang nga kurit
- Kag dapat tama ang haba kag oryentasyon sang mga kurit kag nagakaanggid ang ila kalabaon (batunon ang rectangular prisms)

Kung indi ma-sunod sang sabdyek ang tanan nga instruksyon, indi pag-hatagi sang puntos ang sabdyek sa parte nga ini.

© Z. Nasreddine MD *www.mocatest.org*
Translated by: Aliling NB, Rivera AS, Jamora RD (March 2019)

3. **Pag-Drowing (sang Orasan)[Visuoconstructional Skills (Clock)]**

Instruksyon sa sabdyek: Ipakita ang ikatlo nga lang-at sa tuo kag ihatag ang sumusunod nga instruksiyon: *"Magdrowing ka sang* ***orasan****. Ibutan ang tanan nga numero kag ang oras nga nakabutang diyes (10) ka minutos pagkaligad sang alas onse (11)."*

Instruksyon sa paghatag sang puntos: Isa ka puntos ang ihatag sa kada sumusunod nga tatlo ka pagsulundan:

- Porma (1 punto): Kinahanglan bilog ang porma sang orasan. Pwede batunon ang bilog nga may gamay na distorsyon (e.g., may gamay nga sala sa pagka-sara sang bilog).

- Numero (1 punto): Dapat nakabutang ang tanan nga mga numero kag wala sang gin-dugang nga numero. Dapat tama ang pagkasunod-sunod kag ara sa husto nga lugar palibot sa orasan. Pwede batunon ang Romano nga pagkasulat sang mga numero. Pwede man batunon ang drowing kung ang mga numero ay naka-pwesto sa gwa sang bilog.

- Kamot (1 punto): Kinahanglan duha ka kamot nga nagatabid kag nagatudlo sa husto nga oras. Dapat maathag nga mas malip-ot ang kamot nga nagatudlo sa oras sangsa kamot nga nagatudlo sa minuto. Dapat ibutang ang mga kamot sa tunga sang orasan nga ang ila puno ara sa natung-an sang orasan.

Kung indi ma-sunod ang tanan nga instruksyon, indi pag-hatagi sang puntos ang sa parte nga ini.

4. **Pangalanan [Naming]**

Instruksyon sa Sabdyek: Halin sa wala, itudlo ang kada drowing kag magsiling: *"Isiling mo sa akon ang in inga sapat."*

Instruksyon sa paghatag sang puntos: Hatagan sang isa ka puntos kung tama ang paghatag sang pangalan sa sapat: (1) leon, (2) bukaw, (3) kamelyo.

5. **Memorya [Memory]**

Instruksyon sa Sabdyek: Ang nagsayasat ay may basahon nga lima ka mga salita, isa nga salita kada segundo, ginhatag ang sumusunod nga mga instruksyon: *"Handumanan in inga pagtilaw. May basahon ako nga lima ka mga salita. Pamati ka gid sang maayo kag tandaan mo ang mga basahon ko nga mga salita. Pagkatapos ko basahon ang mga salita, hambalon mo liwat ang mga salita nga ginbasa ko. Indi importante kung ano ang pagkasunod-sunod sang mga salita nga imo ihambal."* Markahan sang tsek ang ginatag nga lang-at sa tagsa ka tinaga nga ginsiling sang sabdyek sa sini nga nauna nga pagtilaw. Kun ang sabdyek nagsiling nga tapos na siya (nadumduman ang tanan nga tinaga), ukon wala'y nadumduman bisan isa ka tinaga, basahon liwat ang listahan nga may sumusunod nga instruksiyon: "*Basahon ko*

© Z. Nasreddine MD *www.mocatest.org*
Translated by: Aliling NB, Rivera AS, Jamora RD (March 2019)

liwat ang pareho nga listahan. Himulatan mo nga madumduman kag isiling mo sa akon ang madamo nga tinaga nga masarangan, kaupod ang mga tinaga nga ginhambal kaina." Butangan sang tsek ang ginhatag nga lang-at sa tagsa ka tinaga nga nadumduman sang sabdyek pagkatapos sang ika-duha nga pagtilaw.
Sa katapusan sang ikaduha nga pagtilaw, ipahibalo sa sabdyek nga pangabayon sa siya nga dumdumon liwat ang ini nga mga tinaga paagi sa pagsiling, *"Pangabayon kita nga dumdumon liwat ang ato nga mga tinaga pagkatapos sang pagtilaw."*

Instruksyon sa paghatag sang puntos: Wala sang ihatag mga puntos para sa Una kag Ikaduha nga Pagtilaw.

6. Atensyon [Attention]

Pasulong nga Pagbasa sang Numero (Forward Digit Span): Pagdumala: Ihatag ang sumusunod nga instruksiyon: *"May basahon ako nga mga numero. Pamatian mo sang maayo kag hambalon mo liwat ang mga ginbasa ko nga mga numero. Kailangan nga tama gid ang pagkasunod-sunod sang mga numero."* Basahon ang lima ka makasunod nga numero nga may kadasigon nga isa ka segundo tagsa isa ka numero.

Pabaliskad nga Pagbasa sang Numero (Backward Digit Span): Pagdumala: Ihatag ang sumusunod nga instruksiyon: *"May basahon ako nga mga numero. Ang himuon mo, hambalon mo liwat ang mga numero nga binasa ko, pero pa-atras."* Basahon ang tatlo ka makasunod nga numero nga may kadasigon nga isa ka segundo tagsa isa ka numero.

Instruksyon sa paghatag sang puntos: Hatagan ang sabdyek sang isa ka puntos ang tagsa husto nga pagliwat sang magkasunod-sunod. Ang husto nga sabat nga 2 – 4 – 7 sa pabaliskad nga pagtilaw.

Pagbantay (Vigilance): Pagdumala: Basahon sang manugsayasat ang nakalista nga letra nga may kadasigon nga isa ka segunda tagsa isa, pagkatapos ang paghatag sang sumusunod nga instruksiyon: *"Mabasa ako sang mga letra. Kung mabatian mo ang letra nga A, tapikon mo isa ka beses ang imo kamot sa la mesa. Indi dapat tapikon ang imo kamot kung indi letra A ang nga mabasa."*

Instruksyon sa paghatag sang puntos: Hatagan sang isa ka puntos kun may sero sa isa ka sayop nga nahimo (kun ang sayop amo ang pagtapik sa indi amo nga letra ukon indi natapik ang letra A).

Serye 7s: Pagdumala: Ginhatag sang manugsayasat ang sumusunod nga instruksiyon: *"Ang himuon mo, ang 100 ibanan mo sang 7. Pagkatapos sina, ang sabat nga makuha mo ibanan mo pagid sang 7 tubtub nga silingon ko ikaw nga mag-untat."* Ihatag sang duha ka beses nag in inga instruksiyon kun kinahanglan.

Instruksyon sa paghatag sang puntos: Ang pinakadako nga puntos nga pwede makuha sa parte nga ini ay 3. Indi maghatag (0) puntos kun wala sang sabat nga husto sa pagbuhin, 1 ka puntos para sa isa ka husto nga pagbuhin, 2 ka puntos para sa duha-tubtub-tatlo nga husto nga pagbuhin, kag 3 puntos kun ang manugpasakop nakasabat sang apat ukon lima nga husto sa pagbuhin. Obserbahan mo ang kada kalkulasyon. Kung magka-sala ang sabdyek sa isa ka kalkulasyon pero tama ang iya nga pag-calcular sa mga naga-sunod nga mga kalkulasyon, pwede gihapon batunon ang mga naga-sunod nga mga tama nga mga kalkulasyon. Halimbawa: Kung ang sabat sang sabdyek ay "92 – 85 – 78 – 71 – 64", indi pag-batunon ang isa ka sala nga "92", pero pwede gihapon batunon ang mga naga-sunod nga mga tama nga mga kalkulasyon. Isa ka sayop ini kag ang in inga butang hatagan sing puntos nga 3.

7. Pagsulit sang dinalan [Sentence Repetition]

Instruksyon sa sabdyek: Mahatag nag manugsayasat sang sumusunod nga instruksiyon: *"May basahon ako sang dinalan. Kailangan kag imo nga hambalon liwat nga nagasibu kung ano ang ginbasa ko* [dulog]: ***Nahibal-an ko lamang nga si Juan ang isa nga nagbulig subong nga adlaw."*** Pagkatapos ang sabat, nagasiling: *"May basahon ako sang isa pa ka dinalan. Kailangan kag imo nga hambalon liwat nga nagasibu kung ano ang ginbasa ko* [dulog]. ***Pirmi gid nagapanago ang kuring sa idalom sang sopa kun ang ido ara sa sulod."***

Instruksyon sa paghatag sang puntos: Hatagan sang 1 ka puntos sa tagsa husto hambalon liwat nga dinalan. Kinahanglan eksakto gid ang pagsulit. Mangin alisto sa mga sayop nga nalipatan (ehemplo, nalikawan ang "lamang", "pirmi") kag pag-ilis/pagdugang (ehemplo, "Si Juan ang isa nga nagbulig sini nga adlaw", pag-ilis sang "nagatago" sa "nagapanago", gin-islan ang plural, etc).

8. Pagkatalunsay sa paghambal [Verbal Fluency]

Instruksyon sa sabdyek: Mahatag ang manugsayasat sang sumusunod nga instruksiyon: *"Isiling sa akon ang mga tinaga sa Hiligaynon nga masarangan mong dumdumon kag nagasugod sa letra nga ara sa letra nga ihambal ko sa imo pagligad ihambal itong instruksiyon. Puwede mo ihambal ang bisan ano nga tinaga nga gusto mo, luas sa mga ngalan (tulad sang Pedro ukon Pangasinan), mga numero, ukon mga tinaga nga sa sugod palang sini pareho ang tunog pero magkalain ang kasugpon, ehemplo, paghigugma, pagpalangga, pag-angga. Ipadulugon ko ikaw pagkatapos sang isa ka minuto. Handa ka na?* [dulog] *Sa isa ka minuto, hatagan mo ako sang mga salita nga naga umpisa sa letra B."* [Orasan sang 60 segundo.] *"Mag-untat."*

Instruksyon sa paghatag sang puntos: Maghatag sang isa ka punto kun ang sabdyek makahatag sang 11 nga mga tinaga ukon madamo nga tinaga sa sulod sang 60 segundo. Ilista ang mga sabat sa idalom ukon sa mga kilid sang papel.

9. Abstraksyon [Abstraction]

Instruksyon sa sabdyek*:* Pangayuon sang manugsayasat sa sabdyek nga ipaathag kun ano ang pagkaanggid sa tagsa-tagsa sang magkaupod nga mga tinaga, halin sa ehemplo nga: *"Isiling sa akon kun paano nangin magkaanggid ang aranghita kag ang saging."* Kun sang sabat yara sa nga konkreto, nagasiling imo liwat: *"Isiling sa akon kun paano nangin magkaanggid pa ang duha."* Kun indi maghatag ang sabdyek sang nagakaigo nga sabat (*prutas),* ihambal ang *"Huo, kag parehong prutas ang duha."* Indi gid maghatag sang dugang pa nga mga instruksiyon nga pagpaathag. Pagkatapos sang ehemplo nga pagtilaw, gilayon nga ihambal ang: "*Nian, isiling mo sa akon paano mangin magkaangid ang bisekleta kag ang tren."* Pagkatapos sang sabat, ipahimo gilayon ang ikaduha nga pagtilaw, sa pagsiling nga: *"Nian isiling mo sa akon paano mangin magkaanggid ang ruler kag relo."* Indi gid maghatag sang dugang nga instruksiyon ukon mga palatandaan.

Instruksyon sa paghatag sang puntos: Ang duha ka ulihi nga magkaupod nga butang lamang ang hatagan sang puntos. Hatagan ang sabdyek sang 1 ka puntos sa kada tama nga sabat.

Pwede batunon ang mga sabat nga ini:

a. Tren kag Bisekleta = mga salakyan, mga pangbiyahe, puwede ka magbiyahe sa duha
b. Ruler kag Relo = mga instrumentong pangtakus, ginausar pangtakus.

Indi pagbatunon ang mga sabat nga ini:

a. Tren kag Bisekleta = may mga ruweda
b. Ruler kag Relo = may mga numero

© Z. Nasreddine MD *www.mocatest.org*
Translated by: Aliling NB, Rivera AS, Jamora RD (March 2019)

10. <u>Nagapalantang panumduman [Delayed Recall]</u>

<u>Instruksyon sa sabdyek:</u> Mahatag ang manugsayasat sang sumusunod nga mga instruksiyon: *"Natandaan mo ang lima ka mga salita nga ginpa-tandaan ko sa imo kagina? Ihambal mo sila sa akon."* Magmarka sang tsek (√) sa tagsang hustong tinagang imo madumduman nga wala sang bisan ano nga palatandaan, sa lang-at nga ginhatag.

<u>Instruksyon sa paghatag sang puntos:</u> **Hatagan ang sabdyek sang isa ka puntos sa kada salita nga madumduman niya <u>ng walang palatandaan.</u>**

Opsyonal:

Pagkatapos sang nagapalantang panumduman, pwede hatagan sang mga palatandaan ang sabdyek kung indi niya madumduman ang mga salita. Magmarka sang tsek (√) sa lang-at nga ginbutang kun ang sabdyek makadumdum sang tinaga sa bulig sang sari-sari nga pagpipilian. Ihatag ang tanan nga tinaga nga indi madumduman sa sin inga pamaagi. Kun indi pa madumduman sang sabdyek ang tinaga pagkatapos maghatag sang palatandaan, ihatag sa iya ang sari-saring pagpipilian nga pagtilaw, paagi sang sumusuno nga ehemplo nga instruksiyon, *"Sa imo panghunahuna ano an sumusunod nga mga tinaga ang mga ini, ILONG, GUYA, ukon KAMOT?*
Usaron ang sumusunod nga kategorya kag/ukon sari-sari nga pagpipilian na palatandaan para sa tagsa ka tinaga, kun nagakaigo:

GUYA:	<u>Kategorya palatandaan:</u> parte ini sang katawan sang tawo	<u>Sari-sari nga pagpilian:</u> ilong, guya, kamot
ASUL:	<u>Kategorya palatandaan:</u> kulay ini siya	<u>Sari-sari nga pagpilian:</u> pula, asul, berde
SIMBAHAN:	<u>Kategorya palatandaan:</u> tinukod ini siya	<u>Sari-sari nga pagpilian:</u> simbahan, eskwelahan, ospital
ROSAS:	<u>Kategorya palatandaan</u>: bulak ini siya	<u>Sari-sari nga pagpilian:</u> rosas, sampaguita, ilang-ilang
SEDA:	<u>Kategorya palatandaan:</u> tela ini siya	<u>Sari-sari nga pagpilian:</u> maong, seda, pranela

<u>Instruksyon sa paghatag sang puntos:</u> **Walang puntos nga ihatag para sa mga nadumduman nga tinaga nga may palatandaan.** Ang mga palatandaan ay ginagamit lamang para sa pagkuha sang impormasyon nga pwede gamiton para mahibal-an kung ano sahi sang problema sa memorya. Para sa mga may kulang sa memorya nga may problema sa pagdumdom, puwede man pauswagon pa ang paghimo paagi sa palatandaan. Para sa kulang nga memorya nga may problema sa pagpasulod sang impormasyon, indi mapa-uswag ang paghimo paagi sa palatandaan.

© Z. Nasreddine MD *www.mocatest.org*

11. Oryentasyon [Orientation]

Instruksyon sa sabdyek: Mahatag ang manugsayasat sang sumusunod nga mga instruksiyon: *"Isiling mo sa akon ang petsa subong"*. Kung ang sabdyek indi naghatag sang kompleto nga sabat, madali nga ihambal ang: *"Isiling sa akon ang [tuig, bulan, eksakto nga adlaw sang ini nga domingo]."* Tungod sini ihambal: *"Nian, isiling sa akon ang ngalan sang sini nga lugar, kag sa ano nga siyudad kita subong."*

Instruksyon sa paghatag sang puntos: Hatagan sang 1 ka puntos sa kada tama nga sabat. Kinahanglan ihambal sang sabdyek ang nagakaigo nga petsa kag ang nagakaigo nga lugar (ngalan sang ospital, klinika, opisina). Wala puntos nga ihatag kun ang sabdyek nakahimo sang sayop sang isa ka adlaw para sa adlaw kag petsa.

KABILUGAN NGA PUNTOS: Sumahon ang lista sang nga puntos nga ara sa tuo nga kilid. Dugangan 2 puntos kun 7 tuig ukon may manubo ang tinun-an, ukon 1 punto kun 8-12 tuig ang tinun-an. Ang pinakadako nga puntos nga pwede makuha sang isa ka sabdyek sa MoCA-Hil ay 30. Ang katapusang puntos sang 22 o mas mataas pa nga normal.

© Z. Nasreddine MD *www.mocatest.org*
Translated by: Aliling NB, Rivera AS, Jamora RD (March 2019)
